# Excessive Consumption of Green Tea as a Risk Factor for Periodontal Disease among Korean Adults

**DOI:** 10.3390/nu8070408

**Published:** 2016-07-02

**Authors:** Kyungdo Han, Eunkyung Hwang, Jun-Beom Park

**Affiliations:** 1 Department of Biostatistics, College of Medicine, The Catholic University of Korea, Seoul 06591, Korea; hkd917@naver.com; 2 Bangmok College of General Education, Myongji University, Seoul 03674, Korea; hwangek@mju.ac.kr; 3 Department of Periodontics, College of Medicine, The Catholic University of Korea, Seoul 06591, Korea

**Keywords:** epidemiology, health surveys, nutrition surveys, oral health, periodontitis, tea

## Abstract

This study was performed to assess the relationship between the amount of green tea that is consumed and periodontitis. It is based on data obtained from the Korea National Health and Nutrition Examination Survey, conducted between 2008 and 2010. A community periodontal index equal to code 3 was defined as moderate periodontitis, and code 4 was defined as severe periodontitis (*n* = 16,726). Consumption of green tea less than one cup per day was associated with a decreased prevalence of periodontal disease among Korean adults. The association between the consumption of green tea and periodontal disease was independent of various potential confounding factors, such as age, sex, body mass index, smoking, drinking, exercise, metabolic syndrome, frequency of tooth brushing per day, use of secondary oral products, the number of dental examination per year, diabetes, hypertension, and white blood cell count. Adjusted odds ratio and 95% confidence interval of no consumption was 1.360 (1.156, 1.601) when participants with consumption of two times per week ≤ x < 7 times per week was considered as a reference. However, consumption of one or more cups per day increased the prevalence of moderate and severe periodontitis. In conclusion, excessive consumption of green tea may be considered as a risk factor for periodontal disease among Korean adults.

## 1. Introduction

Green tea is a currently popular beverage and the use of green tea extracts as dietary supplements for a wide range of applications is increasing due to its health-promoting effects [1]. Green tea contains polyphenols (the major component in green tea), which represent a cluster known as catechins [2]. Catechins are reported to exhibit antioxidant, antimicrobial, anticollagenase, antimutagenic, and chemopreventive properties [3]. Among the polyphenols, epigallocatechin gallate and epicatechin gallate are the most predominant catechins [3]. It is reported that the consumption of green tea reduced the risk of many modern diseases [4]. Green tea has shown a preventive effect against cancer and cardiovascular disease in experimental and epidemiologic studies and within other physiological functions, such as an anti-hypertensive effect, body weight control, antibacterial and antivirasic activity, solar ultraviolet protection, bone mineral density increase, anti-fibrotic properties, and the provision of neuroprotective power [5,6,7].

Moreover, growing evidence suggests a beneficial role of green tea in oral health [8,9]. It has been reported that green tea protects against bacterial-induced dental caries [8]. One previous report showed the association between the consumption of green tea and periodontal disease [10,11]. Green tea extract inhibited the onset of periodontal destruction (loss of attachment and alveolar bone resorption) in experimental periodontitis among rats [10]. Previous reports regarding human participants showed that there was a modest inverse association between the intake of green tea and periodontal disease [11]. However, the effects of the amount of green tea that is consumed and periodontal disease is not well documented. It was hypothesized that there was no significant association between the amount of green tea consumed and periodontal disease. Thus, using nationally representative data, this study was performed to assess the relationship between the amount of green tea consumed and the incidence of periodontitis.

## 2. Materials and Methods 

### 2.1. Overview of the Survey and Participants

This study was based on data obtained from the Korea National Health and Nutrition Examination Survey (KNANES), which was conducted between 2008 and 2010 by the Division of Chronic Disease Surveillance under the Korea Centers for Disease Control and Prevention and the Korean Ministry of Health and Welfare. KNHANES surveys are conducted annually by using a rolling sampling design that involves a complex, stratified, multistage probability-cluster survey of a large representative sample of the non-institutionalized civilian population in South Korea. The present cross-sectional analysis was restricted to adult participants ≥19 years of age that completed the health examination assessment and the nutrition survey, having no missing values for the consumption of green tea or outcome variables. Initially, 29,235 total participants were screened, and the 7424 subjects aged <19 were excluded. After removing 5085 subjects who had missing parameters, 16,726 total subjects were included. The consumption of green tea, carbonated beverages, and coffee was calculated according to the survey. Information regarding age, residence area, income, occupation, education, exercise, smoking history, and alcohol intake was collected during the health interview. All the individuals provided written informed consent. This study was approved by the Institutional Review Board of the Korean Center for Disease Control and Prevention and conducted according to the Helsinki Declaration-based Ethical Principles for Medical Research Involving Human Subjects.

Trained inspectors obtained the anthropometric evaluation. Body weight and height were measured to the nearest 0.1 kg and 0.1 cm, respectively, with participants wearing light indoor clothing without shoes. Body mass index was calculated as body weight (kg) divided by the squared height (m^2^), and waist circumference was measured at the narrowest point between the lower border of the ribcage and the iliac crest in a standing position. Income level was categorized into four quartile groups, the lowest of which included households with a monthly income of <$1092.40 USD [12]. The participants’ education level was categorized into two groups: below high school and high school graduates or above. Smoking status was divided into two categories: current smoker or not. Smoking status was defined according to self-reported cigarette use and based on current smoking habits [13]. Alcohol consumption was assessed by asking the participants about their drinking behavior during the month prior to the interview. The responses were converted into the amount of pure alcohol (in grams) consumed per day by using the average number of alcoholic beverages consumed and the frequency of alcohol consumption. Alcohol consumption status was categorized into three groups according to average daily alcohol consumption: nondrinkers, and light to moderate (1–30 g), and heavy (>30 g) drinkers [14]. Regular exercise was defined as engaging in a moderate fitness activity on a regular basis for ≥30 min at a time, at least five times per week, or for ≥20 min at a time at least three times per week in a vigorous fitness activity. Daily energy, fat, protein, and calcium intake were calculated using a food database developed for the KNHANES and the food composition table published by the National Rural Living Science Institute under the Rural Development Administration.

### 2.2. Biochemical Measurements and the Definition of Metabolic Syndrome

To measure concentrations of serum-fasting plasma glucose and high-density lipoprotein cholesterol and white blood cell count, a blood sample was collected from the antecubital vein of each participant after he or she fasted for more than 8 h. Blood samples were analyzed within 24 h of transportation. Levels of serum-fasting plasma glucose, total cholesterol, triglycerides, and high-density lipoprotein cholesterol were measured with a Hitachi Automatic Analyzer 7600 (Hitachi, Tokyo, Japan) by enzymatic methods using commercially available kits (Daiichi, Tokyo, Japan). White blood cell count was measured from the blood sample. 

A standard mercury sphygmomanometer (Baumanometer, W.A. Baum Co., Copiague, NY, USA) was used to acquire a blood pressure measurement. Systolic blood pressure and diastolic blood pressure were measured twice at five-minute intervals, and the average values were used for the analysis. 

Metabolic syndrome was defined according to the American Heart Association/National Heart, Lung, and Blood Institute Scientific Statement criteria for Asians [15,16]. Diabetes is defined as fasting blood sugar greater than 126 mg/dL or when the individual was currently using anti-diabetic medications [17]. Hypertension was defined as an average BP of ≥140/90 mmHg or if the individuals were taking antihypertensive medication [18,19]. 

### 2.3. Periodontal Treatment Needs and Oral Health Behaviors

The frequency of daily tooth brushing was measured by the total number of times the teeth were brushed per day. Dental checkup within a year, self-reported oral status, chewing ability, and speech were evaluated. 

The World Health Organization’s community periodontal index (CPI) was used to assess periodontal treatment needs and defined periodontal disease. All teeth of each subject were divided into sextants. Ten specific index teeth (17, 16, 11, 26, 27, 36, 37, 31, 46, and 47) were examined to evaluate each sextant’s score. A sextant was only scanned if two or more teeth were present. If index teeth were absent from a sextant, all of the remaining teeth were examined to produce the score, and the highest score was recorded as the score for the sextant. The CPI score represented periodontitis status as follows—0: normal, 1: gingival bleeding, 2: calculus, 3: a shallow pocket with depth of 3.5–5.5 mm or moderate periodontitis, and 4: a deep pocket depth ≥5.5 mm or severe periodontitis [20]. A CPI of 3 or 4 was considered as having periodontitis (moderate and severe periodontitis, respectively) [14]. In 2008 KNHANES, 24 trained dentists examined the periodontal status of subjects. Twenty-nine and 36 dentists performed periodontal examination in 2009 and 2010 KNHANES, respectively.

### 2.4. Statistical Analyses

All data are presented as the mean ± standard error or as a percentage (standard error). Logarithmic transformation was performed to achieve normal distribution when necessary. Student’s *t*-test or the chi-square test was used to investigate the differences in the presence of periodontal treatment needs according to the variables. Survey sample weights were used in all analyses to produce estimates that were representative of the non-institutionalized civilian Korean population. A statistical software package (SAS version 9.2 for Windows, SAS Institute, Cary, NC, USA) was used for statistical analysis to account for the complex sampling design. Two-sided *p* values of < 0.05 were considered statistically significant. Multiple logistic regression analyses were used to assess the associations of periodontal treatment needs and the consumption of green tea after covariate adjustment. The covariates were age, sex, body mass index, smoking, drinking, exercise, metabolic syndrome, frequency of tooth brushing per day, use of secondary oral products, number of dental examinations within a year, diabetes, hypertension, and white blood cell count. 

## 3. Results

Table 1 describes the baseline characteristics of the study individuals according to the presence of periodontal disease. The mean age, body mass index, and waist circumference were significantly higher in participants with periodontal disease. The percentage of individuals with periodontitis was higher in current smokers and drinkers. 

Table 2 shows the subgroup analysis regarding the consumption of green tea by oral health. Self-reported oral status showed that the individuals with problems were lowest with the group of two times per week ≤ x < 7 times per week. The individuals with chewing discomfort were lowest with the group of two times per week ≤ x < 7 times per week.

The adjusted odds ratio, 95% confidence interval and *P* value of the prevalence of periodontitis in multivariate logistic regression model for the consumption of green tea is shown in Table 3. The odd ratio (95% confidence intervals) of the prevalence of moderate and severe periodontitis was 1.360 (1.156, 1.601), 1.262 (1.047, 1.522), 1.092 (0.912, 1.309), 1, and 1.375 (1.135, 1.666) for no consumption, ≤once per month, two times per month ≤ x < 2 times per week, two times per week ≤ x < 7 times per week, and ≥once per day, respectively (Table 3). 

## 4. Discussion

This study evaluated the relationship between the amount of green tea consumed and the incidence of periodontitis with nationally representative data. This study showed that consumption of less than one cup of green tea per day was associated with a decrease in the prevalence of periodontal disease among Korean adults. This study has also shown that consumption of one cup or more increased the prevalence of periodontitis. This association between the consumption of carbonated beverages and periodontal disease was independent of various potential confounding factors, such as age, sex, body mass index, smoking, drinking, exercise, metabolic syndrome, frequency of tooth brushing per day, use of secondary oral products, number of dental examination within a year, diabetes, hypertension, and white blood cell count. 

Green tea modulates cytokine expression in the periodontium and attenuates alveolar bone resorption in type 1 diabetic rats [21]. In the previous report, green tea intake was reported to reduce the expression of the pro-inflammatory cytokine tumor necrosis factor-α and the osteoclastogenic mediator receptor activator of nuclear factor kappa-B ligand to normal levels, while increasing the expression of the anti-inflammatory cytokine interleukin-10, the osteogenesis-related factor runt-related transcription factor 2, and the anti-osteoclastogenic factor osteoprotegerin [21]. Green tea is shown to inhibit the growth and cellular adherence of bacterial pathogens and their production of virulence factors [11].

Various methods have been applied for the delivery of the green tea extracts. Green tea extract was incorporated into hydroxylpropyl methylcellulose [22,23]. This system showed a bactericidal effect against Gram-negative anaerobic rods and was effective in improving periodontal status. Adjunctive local drug therapy with thermo-reversible sustained-release green tea gel has been shown to reduce inflammation and pockets of the clinical trial in patients with chronic periodontitis [24]. It has been suggested that local drug delivery using green tea extract could be used as an adjunct in the treatment of chronic periodontitis [22,25,26]. Green tea has been added to dentifrice in previous studies [27,28]. In a previous study, dentifrice containing green tea extracts showed a greater reduction in gingival inflammation and improved periodontal parameters in comparison with fluoride-triclosan dentifrice [28]. Comparative evaluation of the anti-plaque effectiveness of green tea catechin mouthwash was performed with chlorhexidine gluconate; green tea catechin mouthwash (0.25%) and chlorhexidine mouthwash (0.12%) have shown comparable results in plaque reduction [29]. Collectively, it was suggested that the local delivery of green tea catechin, along with scaling and root planning, is more effective than scaling and root planing alone [25,26].

This study showed that drinking less than one cup of green tea per day produced beneficial effects on periodontal health. The beneficial effect of the consumption of green tea on periodontitis may be partially explained by the following. The green tea extract of epigallocatechin gallate exerts potent protective effects against oxidative stress, inflammation, protein aggregation, autophagy, and cell death in vitro as well as in vivo [30,31]. Catechin epigallocatechin gallate is reported to destroy established *Porphyromonas gingivalis* biofilms and inhibit biofilm formation [32,33]. Epigallocatechin gallate is shown to inhibit the production of C-C motif chemokine ligand 11 [34]. Epigallocatechin gallate is shown to diminish cysteine-rich 61 (a potential osteolytic mediator) expression in osteoblasts and, subsequently, reduce macrophage chemotaxis into the lesions by diminishing C-C motif chemokine ligand 2 (a chemokine responsible for macrophage chemotaxis) [9]. In oral epithelial cells and fibroblasts, epigallocatechin gallate showed a neutralizing effect on nicotine-induced toxicity [35]. In a three-dimensional co-culture model of gingival epithelial cells and fibroblasts, epigallocatechin-3-gallate reduced the lipopolysaccharide-induced inflammatory response [36]. Among green tea catechins, epigallocatechin gallate and epicatechin gallate showed the most potent inhibitory effect on collagenase activity when catechins were added to a reaction mixture containing collagenase and collagen [37].

This study has also clearly shown that excessive consumption of green tea increases the risk of periodontitis. The harmful effect of the excessive consumption of green tea on periodontitis may be partially explained by the following. It was reported that a cup of tea contains 15 mg and excessive amounts of green tea consumption will lead to significant amount of caffeine consumption [38]. Caffeine is shown to increase bone loss and enhance the progression of periodontitis [39], and caffeine intake is considered to be one of the possible risk factors for periodontitis [40]. The temperature of the green tea may damage the mucosa or accelerate metabolic reactions, including hastening the absorption of harmful substances in cigarette and alcohol [41]. A previous report has shown that concentrated green tea extract induces severe acute hepatitis and has suggested that such concentrated herbal extracts from green tea may not be free of adverse effects under certain circumstances, including the excessive consumption of green tea [1]. 

These limitations should be considered. Because of this study’s cross-sectional design, the causal direction of the associations between the consumption of green tea and periodontal disease cannot be ascertained [13]. Another limitation of this study is that individual consumption habits were obtained on a recall basis [12]. Other drinks including coffee and carbonated beverages should also be considered for the risk evaluation. However, the data used in this study were collected from the KNHANES, a nationally representative sample, whose participants were recruited using a multi-stage clustered probability design. Further, survey sample weights adjusted for participation rate and response rate were used for all analyses to consider selection bias and to serve as nationally representative data [42]. The association between the consumption of green tea and periodontitis were evaluated using multiple logistic regression analyses after adjusting for confounding factors [13]. Thus, the results from this study can be considered reliable and meaningful to the general audience.

## 5. Conclusions 

The results generally indicated a negative association between the consumption of green tea and the risk of periodontal disease among Korean adults. However, the consumption of one or more cups per day increased the risk of moderate and severe periodontitis. In conclusion, the consumption of green tea may be considered an independent risk indicator of periodontal disease among Korean adults, and we suggest that good periodontal health may benefit from moderate consumption of green tea (less than one cup per day).

## Figures and Tables

**Table 1:** Baseline characteristics of study individuals according to severity of periodontal disease.

	Periodontal Disease (Moderate (CPI 3) and Severe Periodontitis (CPI 4))		
	No	Yes	*p*-Value *
Unweighted *n*	11290	5436	
**Consumption of green tea**			<0.0001
No consumption	35.2 (0.7)	44.8 (1)	
≤Once per month	15.4 (0.4)	13.4 (0.6)	
Two times per month ≤ x< 2 times per week	18.9 (0.5)	14.9 (0.6)	
Two times per week ≤ x < 7 times per week	16.2 (0.5)	11.9 (0.6)	
≥Once per day	14.3 (0.4)	15 (0.7)	
Age (years)	40.57 ± 0.26	52.08 ± 0.31	<0.0001
Sex (male)	45.7 (0.5)	59.4 (0.7)	<0.0001
Body mass index (kg/m^2^)	23.4 ± 0.04	24.13 ± 0.06	<0.0001
White blood cell (×10^9^/L) **	5.87 (5.91–5.91)	6.21 (6.26–6.26)	<0.0001
Energy intake (kcal/day)	2015.17 ± 12.08	1997.59 ± 17.14	0.3523
Calcium intake (mg/day)	513.4 ± 4.28	518.12 ± 7.45	0.5757
Smoking	22.7 (0.5)	30.8 (0.8)	<0.0001
**Drinking**			<0.0001
Non-drinker	21.8 (0.5)	27 (0.7)	
Mild to moderate drinker	68.9 (0.6)	60.7 (0.8)	
Heavy drinker	9.3 (0.4)	12.3 (0.6)	
Exercise (yes)	24.9 (0.6)	26.7 (0.8)	0.0417
Income (the lowest quartile)	13.5 (0.6)	19.9 (0.8)	<0.0001
Education (high school graduate or higher)	78.4 (0.6)	55.8 (1.2)	<0.0001
Residence (urban)	83.1 (1.5)	76.2 (2.1)	<0.0001
Occupation (yes)	61.5 (0.6)	65.9 (0.9)	<0.0001
Diabetes	5.5 (0.2)	13.8 (0.6)	<0.0001
Hypertension	20.8 (0.5)	38.9 (0.9)	<0.0001
Metabolic syndrome	20.2 (0.5)	38 (0.9)	<0.0001
**Consumption of coffee**			<0.0001
No consumption	12.9 (0.4)	11.1 (0.5)	
≤Once per month	4.7 (0.3)	3.4 (0.3)	
Two times per month ≤ x < 2 times per week	7.7 (0.3)	6.4 (0.4)	
Two times per week ≤ x < 7 times per week	13.0 (0.4)	10.7 (0.5)	
≥Once per day	61.7 (0.6)	68.4 (0.8)	
**Consumption of carbonated beverages**			<0.0001
No consumption	33.5 (0.6)	44.5 (1)	
≤Once per month	22.9 (0.5)	24.3 (0.7)	
Two times per month ≤ x < 2 times per week	25.4 (0.5)	20.3 (0.7)	
Two times per week ≤ x < 7 times per week	15.4 (0.5)	9.7 (0.6)	
≥Once per day	2.8 (0.2)	1.2 (0.2)	
**Frequency of tooth brushing per day**			<0.0001
≤1	10 (0.4)	15.8 (0.7)	
2	42.4 (0.7)	46.5 (0.9)	
≥3	47.6 (0.7)	37.6 (1.0)	
Use of secondary oral products	44.2 (9.8)	33.0 (1.1)	<0.0001
Dental checkup within a year (yes)	27.6 (0.8)	25.6 (0.9)	0.0414
**Self-reported oral status**			<0.0001
Favorable	13.8 (0.4)	8.8 (0.5)	
Average	44.6 (0.6)	31.7 (0.9)	
Problematic	41.6 (0.6)	59.5 (1)	
**Chewing**			<0.0001
Discomfort	20.7 (0.5)	41.1 (0.9)	
Minor problem	15.3 (0.5)	15.5 (0.7)	
No discomfort	64 (0.6)	43.4 (0.9)	
**Speech**			<0.0001
Discomfort	5.7 (0.3)	12 (0.5)	
Minor problem	6.3 (0.3)	9.8 (0.5)	
No discomfort	88 (0.4)	78.1 (0.8)	

Data are presented as mean ± standard error or percentages (standard error). * *p* values were obtained by an independent *t*-test for continuous variables or the chi-square test for categorical variables. ** Log transformation was applied to the value, and the geometric mean (95% confidence of interval) is shown.

**Table 2:** Baseline characteristics of study individuals according to severity of periodontal disease.

Consumption of Green Tea	No Consumption	≤Once per Month	Two Times per Month ≤ x < 2 Times per Week	Two Times per Week ≤ x < 7 Times per Week	≥Once per Day	*p*-Value
**Frequency of tooth brushing per day**						<0.0001
≤1	15.6 (0.6)	10.9 (0.8)	10.1 (0.7)	8.4 (0.8)	7.8 (0.7)	
2	46.4 (0.8)	47.6 (1.2)	42.6 (1.1)	38.9 (1.3)	38.4 (1.3)	
≥3	38 (0.8)	41.4 (1.3)	47.3 (1.2)	52.6 (1.3)	53.8 (1.3)	
Dental checkup within a year (yes)	22.9 (0.9)	25.5 (1.1)	28.1 (1.2)	32.5 (1.2)	32.3 (1.3)	<0.0001
**Self-reported oral status**						<0.0001
Favorable	11.1 (0.5)	11.3 (0.8)	13.9 (0.8)	14.8 (0.9)	12.3 (0.8)	
Average	39.4 (0.8)	41.6 (1.2)	41.7 (1.1)	41.3 (1.3)	42.3 (1.3)	
Problematic	49.5 (0.9)	47.1 (1.2)	44.4 (1.2)	43.9 (1.3)	45.4 (1.3)	
**Chewing**						<0.0001
Discomfort	32.8 (0.8)	23.9 (1)	23.1 (1)	20.3 (1)	24.4 (1.2)	
Minor problem	14.7 (0.6)	15.8 (0.9)	15.7 (0.9)	15.5 (0.9)	16.1 (1)	
No discomfort	52.5 (0.8)	60.2 (1.2)	61.3 (1.2)	64.2 (1.3)	59.5 (1.3)	
**Speech**						<0.0001
Discomfort	10.3 (0.5)	6.3 (0.6)	6.6 (0.5)	5.5 (0.5)	5.1 (0.5)	
Minor problem	8.4 (0.4)	7.1 (0.7)	6.4 (0.5)	6.1 (0.6)	7.0 (0.7)	
No discomfort	81.3 (0.6)	86.6 (0.8)	87 (0.7)	88.4 (0.8)	87.9 (0.8)	

Data are presented as percentages (standard error).

**Table 3:** Adjusted odds ratio, 95% confidence interval and *P* value of the prevalence of periodontitis in multivariate logistic regression model for the consumption of green tea.

Consumption of Green Tea	Model 1	Model 2	Model 3
No consumption	1.291 (1.12, 1.488)	1.256 (1.086, 1.452)	1.359 (1.155, 1.600)
≤Once per month	1.094 (0.939, 1.275)	1.098 (0.939, 1.285)	1.264 (1.048, 1.524)
Two times per month ≤ x < 2 times per week	1.035 (0.891, 1.202)	1.03 (0.884, 1.201)	1.089 (0.909, 1.304)
Two times per week ≤ x < 7 times per week	1	1	1
≥Once per day	1.31 (1.11, 1.546)	1.289 (1.09, 1.525)	1.380 (1.139, 1.672)
*P* for trend	0.0003	0.0019	0.00065

Model 1: Age and sex adjusted; Model 2: Model 1 plus body mass index, smoking, drinking and exercise adjusted; Model 3: Model 2 plus metabolic syndrome, frequency of tooth brushing per day, use of secondary oral products and white blood cell adjusted.

## Abbreviations

The following abbreviations are used in this manuscript:

KNANES	Korea National Health and Nutrition Examination Survey
